# Atopic dermatitis and risk of gastroesophageal reflux disease: A nationwide population-based study

**DOI:** 10.1371/journal.pone.0281883

**Published:** 2023-02-17

**Authors:** Seung Won Lee, Jiwon Park, Hayeon Kim, Yong Woo Jung, Yoo Sang Baek, Yejee Lim, Kyungim Kim

**Affiliations:** 1 Institute of Pharmaceutical Science, Korea University, Sejong, South Korea; 2 College of Pharmacy, Korea University, Sejong, South Korea; 3 Department of Dermatology, Guro Hospital, Korea University College of Medicine, Seoul, South Korea; 4 Department of Internal Medicine, Seoul National University Bundang Hospital, Seongnam, South Korea; National Skin Centre, SINGAPORE

## Abstract

**Background:**

As atopic dermatitis (AD) has been found to be related to various comorbidities as well as substantial patient burden, questions of a possible relationship between AD and nonallergic diseases beyond allergic diseases have also been raised.

**Objective:**

The aim of this nationwide matched cohort study was to evaluate whether AD would increase the development of gastroesophageal reflux disease (GERD).

**Methods:**

Patients diagnosed with AD were identified from the National Health Insurance Service-National Sample Cohort (NHIS-NSC) 2.0 database in South Korea from 2002 to 2015. Finally, 9,164 adults with AD (≥20 years old) and age, sex, household income, region of residence, disability, and baseline year-matched 9,164 controls were included in the analysis. Hazard ratio (HR) with 95% confidence interval (CI) for the development of GERD was estimated using a Cox proportional hazard regression model.

**Results:**

Overall, 12.3% of the patients in the AD group developed GERD, whereas 10.4% of the individuals in the control group developed GERD. The results of the adjusted model revealed that patients with AD had a significantly increased risk of developing GERD (adjusted HR, 1.15; 95% CI, 1.06–1.26) compared with the matched controls. Increased risk of developing GERD was consistent in subgroup analyses by sex or age groups under 60 years old as well as all the sensitivity analyses performed.

**Conclusions:**

This study suggested that appropriate management should be considered in adults with AD to prevent GERD, because AD was found to be associated with an increased risk of subsequent GERD.

## Introduction

Atopic dermatitis (AD) is one of the common chronic, inflammatory skin diseases, with approximately 230 million people estimated to be affected worldwide [1–3]. Although AD has traditionally been considered a childhood disease, recent epidemiological studies have shown that the prevalence of AD in adults is approximately 5%–10%, and its prevalence is increasing worldwide [4–7]. The pathophysiology of AD is complex, and AD is regarded to occur through a combination of genetic, environmental, and immunologic factors. In addition, as the activation of skin and systemic immunity has been suggested to occur in AD, interest has expanded to nonallergic conditions beyond the allergic diseases as comorbidities of AD [8–12].

Gastroesophageal reflux disease (GERD) is a common gastrointestinal disorder that includes symptoms and complications resulting from the reflux of the stomach contents into the esophagus [13]. The prevalence of GERD is increasing worldwide, with currently 15%–25% prevalence in high-income countries and <10% in most low- and middle-income countries [14–16]. It has been suggested that GERD is associated with some esophageal and extraesophageal diseases, such as reflux-induced cough, laryngitis, asthma, and dental erosion, but it is difficult to explain a causal correlation between GERD and these esophageal and extraesophageal diseases because there may be a bidirectional effect [17, 18].

Both AD and GERD are highly prevalent and well-known diseases that impair the health-related quality of life of patients [15, 19]; however, few studies have evaluated the impact of AD on GERD. Understanding the link between AD and GERD may help lessen the associated burden of these comorbidities in patients with AD. Thus, this study aimed to evaluate the risk of developing GERD in adult patients newly diagnosed with AD using well-established national cohort data in Korea.

## Materials and methods

### Data source

This retrospective propensity score matching (PSM) cohort study used data from the National Health Insurance Service-National Sample Cohort (NHIS-NSC) 2.0 database of the National Health Insurance Service (NHIS) from 2002 to 2015. This NHIS is the compulsory single-payer national health care coverage system in South Korea. The NHIS-NSC is a large-scale, population-based cohort data consisting of an approximately 2.2% representative sample of the general Korean population. Sampling consisted of a systematic stratified random sample with proportional allocation within each stratum. The database contains longitudinal health-related information including socio-demographics, disease diagnoses [International Classification of Disease, Tenth Revision (ICD‐10)], therapeutic procedures, and drug prescriptions (brand name, generic name, prescription date, days of supply, dose and route of administration), type of medical utilization (outpatient, inpatient, or emergency department), and annual or biennial national health screening examinations that include health questionnaire surveys, physical examinations, and laboratory test [20]. A detailed description of these data has been reported elsewhere [21].

This study was approved by the Institutional Review Board of Korea University (KUIRB-2021-0201-01) and the Korea NHIS National Health Information Data Request Review Committee (NHIS-2021-2-212). All methods were performed in accordance with the approved guidelines and regulations. Informed consent was waived due to the retrospective nature of this study.

### Study population

**Fig 1:**
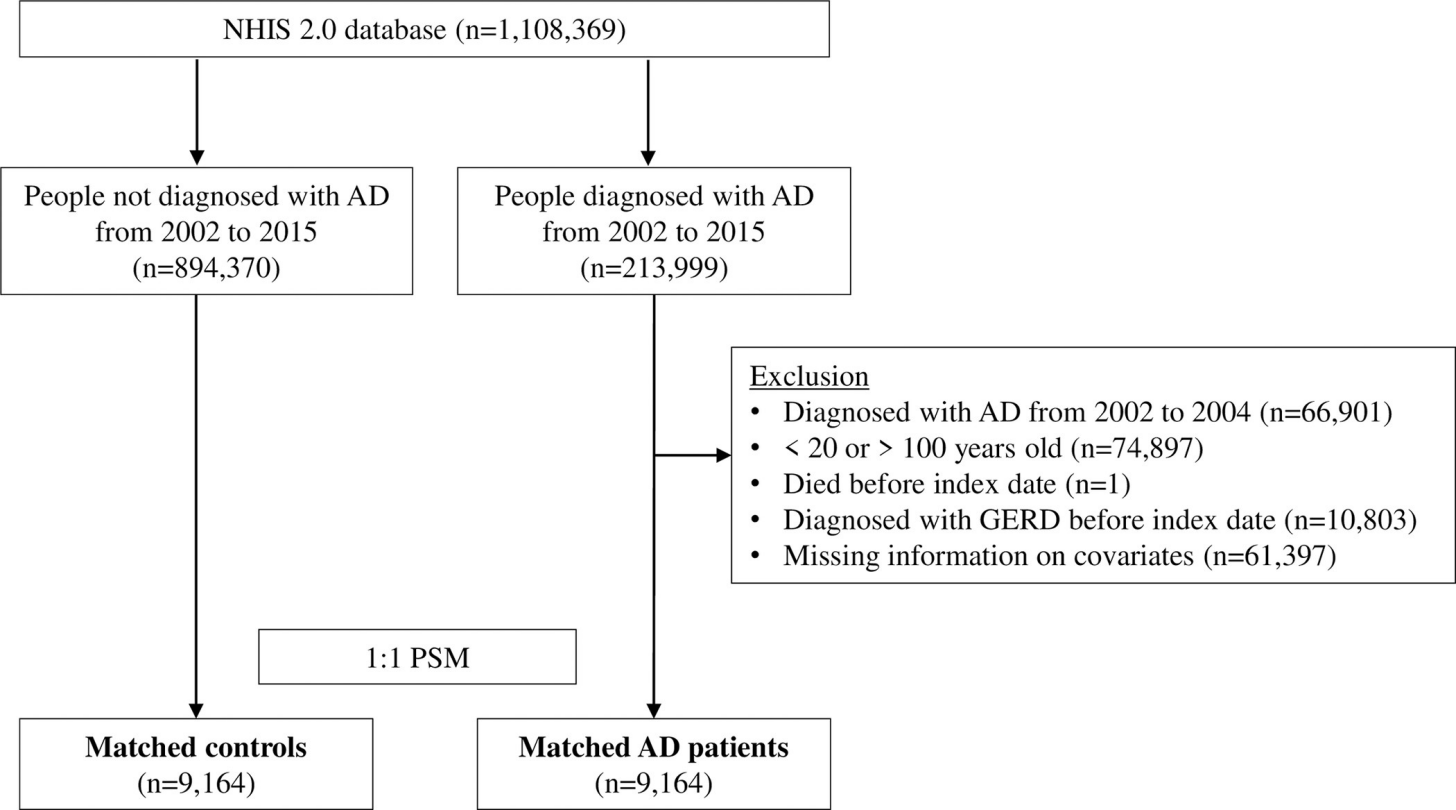
Flow diagram of the study population. AD, atopic dermatitis; GERD, gastroesophageal reflux disease; NHIS, National Health Insurance Service; PSM, propensity score matching.

Patients with AD were defined as those having received at least one diagnostic code (ICD-10 code L20) and prescribed AD-related medications on the same day to reduce the probability of misclassification. AD-related medications included topical or oral corticosteroids, immunosuppressants (methotrexate, cyclosporine, mycophenolate mofetil, or azathioprine), topical calcineurin inhibitors (tacrolimus or pimecrolimus), or antihistamines. The date of the first diagnosis of AD for each patient was defined as the index date. The exclusion criteria were patients under 20 or over 100 years old, who died or were diagnosed with GERD before the index date, or who had incomplete information on covariates. In addition, patients diagnosed with AD between 2002 to 2004 were excluded, and this 3-year washout period allowed this study to include new AD cases. Selected AD patients were matched 1:1 with controls who had never been diagnosed with AD from 2002 to 2015 based on the covariates at the AD patient’s index date using PSM. PSM is a widely used method to reduce potential confounders and balance the baseline covariates of the two groups [22]. Propensity scores were derived from the predicted probability of subjects with AD versus without AD using a logistic regression model with adjustment for the following confounders: age, sex, household income, region of residence, disability, and baseline year. To reduce immortal time bias, the individual index date was set to the same date as the AD diagnosis in AD patients and matched controls [23]. The study population selection process is detailed in Fig 1.

Patients with severe AD were defined as those who had received oral corticosteroids or immunosuppressants. Other AD cases were classified as nonsevere [24]. For statistical analysis, severe AD patients were matched 1:1 with nonsevere AD patients based on the covariates of the first date of severe AD using PSM.

### Study outcome and follow-up period

The study outcome of interest was newly onset GERD after the index date. GERD was defined as a patient with two or more diagnoses of GERD (ICD-10 code K21) who had been prescribed GERD-related medications for more than 2 weeks. GERD-related medications included histamine 2-receptor antagonists, proton pump inhibitors, and potassium-competitive acid blockers. Patients with AD and their matched controls were followed up from the index date. Follow-up was terminated on the date of the study outcome, death, or at the end of the study (December 31, 2015), whichever occurred first.

### Covariates

To control for potential confounding factors in the analyses, covariates were identified based on previous studies, expert opinion, and covariate availability within the data. The covariates used in this study included age, sex, household income, region of residence, disability, Charlson comorbidity index (CCI) score, smoking status, body mass index (BMI), co-medications, and baseline year.

The age groups were classified using 10-year intervals. A total of eight groups aged ≥20 years old were included. The household income groups initially provided with 11 classes (class 0, lowest income; class 10, highest income) from the NHIS database were recategorized into three groups (low, class 0–2; medium, class 3–7; high, class 8–10). The region of residence was recategorized into urban (Seoul, Busan, Daegu, Incheon, Gwangju, Daejeon, and Ulsan) and rural (Gyeonggi, Gangwon, Chungcheongbuk, Chungcheongnam, Jeollabuk, Jeollanam, Gyeongsangbuk, Gyeongsangnam, and Jeju). The patients’ CCI scores were estimated from their disease records using previously validated algorithms [25]. Smoking status was categorized as current smoker, ex-smoker, and never smoked. BMI was calculated as an individual’s body weight in kilograms divided by their height in meters squared (kg/m^2^). In this study, BMI was categorized into underweight (BMI less than 18 kg/m^2^), normal (BMI between 18 to 25 kg/m^2^), and overweight (BMI more than 25 kg/m^2^). Co-medications, such as calcium channel blockers (amlodipine, felodipine, flunarizine, isradipine, levamlodipine, nicardipine, nifedipine, nimodipine, nisoldipine, diltiazem, and verapamil), antidepressants (amitriptyline, amoxapine, clomipramine, desipramine, doxepin, imipramine, maprotiline, nortriptyline, protriptyline, and trimipramine), theophylline, and anticholinergic drugs (benztropine, dicyclomine, hyoscyamine, isopropamide, and scopolamine), which are known to contribute to the development of GERD, were also considered as covariates if the medication was used for more than 60 days between the index date and the last observation date.

### Statistical analysis

Baseline characteristics of the AD group and control group were reported as frequencies and percentages, and a Chi-square test was used to compare the general characteristics of these groups. Cox proportional hazards model was used to estimate the hazard ratio (HR) and 95% confidence interval (CI) for GERD in the AD group versus matched control group. The risk of developing GERD in the severe AD group was also compared to the nonsevere AD group. In these analyses, crude and adjusted models for age, sex, household income, region of residence, disability, CCI score, smoking status, BMI, co-medications, and baseline year were used. The cumulative incidence of GERD was estimated using the Kaplan–Meier method and log-rank tests.

Subgroup analyses according to sex and age were conducted. For sensitivity analysis, the definitions of AD or GERD were changed as follows: (1) the definition of AD was changed from at least one diagnosis code and AD-related treatments on the same day to two or more on separate days, (2) the definition of GERD was changed from two or more diagnosis code and GERD-related treatments for more than 2 weeks to three or more diagnosis codes and treatments for more than 3 weeks, and (3) antacids were added to the list of drugs defining GERD. All analyses were conducted using SAS Enterprise Guide 7.1 (SAS Institute Inc., Cary, NC, USA), and statistical significance was defined as a two-sided P value of <0.05.

## Results

### Characteristics of the study population

The present study included each of 9,164 subjects in adult AD and matched control groups after PSM. Table 1 shows the baseline characteristics of the study population after PSM. No significant differences were found between the two groups except for unadjusted variables in PSM. The median follow-up period was 4.2 years and 4.4 years in the AD and control groups, respectively. During the follow-up period, a total of 2,079 GERD cases occurred, including 1,130 (12.3%) and 949 (10.4%) patients in the AD and control groups, respectively.

**Table 1:** Baseline characteristics of the study population after PSM.

Variables	People without AD (n = 9,164)		People with AD (n = 9,164)		*P* value
Sex					1.0000
Male	4,815	(52.54)	4,816	(52.55)	
Female	4,349	(47.46)	4,348	(47.45)	
Age, years					0.8847
20–29	857	(9.35)	857	(9.35)	
30–39	1,700	(18.55)	1,700	(18.55)	
40–49	2,363	(25.79)	2,363	(25.79)	
50–59	2,141	(23.36)	2,141	(23.36)	
60–69	1,337	(14.59)	1,340	(14.62)	
70–79	664	(7.25)	664	(7.25)	
80–89	99	(1.08)	99	(1.08)	
90–100	3	(0.03)	–	(0.00)	
Household income^a^					1.0000
Medium (3–7)	4,864	(53.08)	4,864	(53.08)	
High (8–10)	4,300	(46.92)	4,300	(46.92)	
Region of residence^b^					0.8475
Urban	4,336	(47.32)	4,323	(47.17)	
Rural	4,828	(52.68)	4,841	(52.83)	
Disability	440	(4.80)	428	(4.67)	0.7021
CCI score^c^					<0.0001
0	1,226	(13.38)	798	(8.71)	
1	2,163	(23.60)	1,901	(20.74)	
2	2,121	(23.14)	2,173	(23.71)	
≥3	3,654	(39.87)	4,292	(46.84)	
Smoking status^c^					0.0004
Current smoker	5,696	(62.16)	5,811	(63.41)	
Ex-smoker	1,299	(14.18)	1,398	(15.26)	
Never smoked	2,169	(23.67)	1,955	(21.33)	
BMI, kg/m^2^^c^					0.9838
Underweight (<18)	193	(2.11)	196	(2.14)	
Normal (18–24.9)	5,931	(64.72)	5,923	(64.63)	
Overweight (≥25)	3,040	(33.17)	3,045	(33.23)	
Co-medications^c^					
Calcium channel blockers	1,146	(12.51)	1,202	(13.12)	0.2241
Antidepressants	207	(2.26)	237	(2.59)	0.1635
Theophylline	43	(0.47)	80	(0.87)	0.0011
Anticholinergic drugs	73	(0.80)	101	(1.10)	0.0397

Variables are presented as numbers (%), and the *P* value was derived from the Chi-square test.

AD, atopic dermatitis; BMI, body mass index; CCI, Charlson comorbidity index; GERD, gastroesophageal reflux disease; PSM, propensity score matching.

^a^Household income was originally divided into 11 classes (class 0, lowest income; class 10, highest income) in the database and was reclassified into 3 groups (low, class 0–2; medium, class 3–7; high, 8–10). After PSM, no subjects were in the “low (class 0–2)” group.

^b^Region of residence was recategorized into urban (Seoul, Busan, Daegu, Incheon, Gwangju, Daejeon, and Ulsan) and rural (Gyeonggi, Gangwon, Chungcheongbuk, Chungcheongnam, Jeollabuk, Jeollanam, Gyeongsangbuk, Gyeongsangnam, and Jeju).

^c^Unadjusted variables in PSM

### Association of AD with GERD

**Fig 2:**
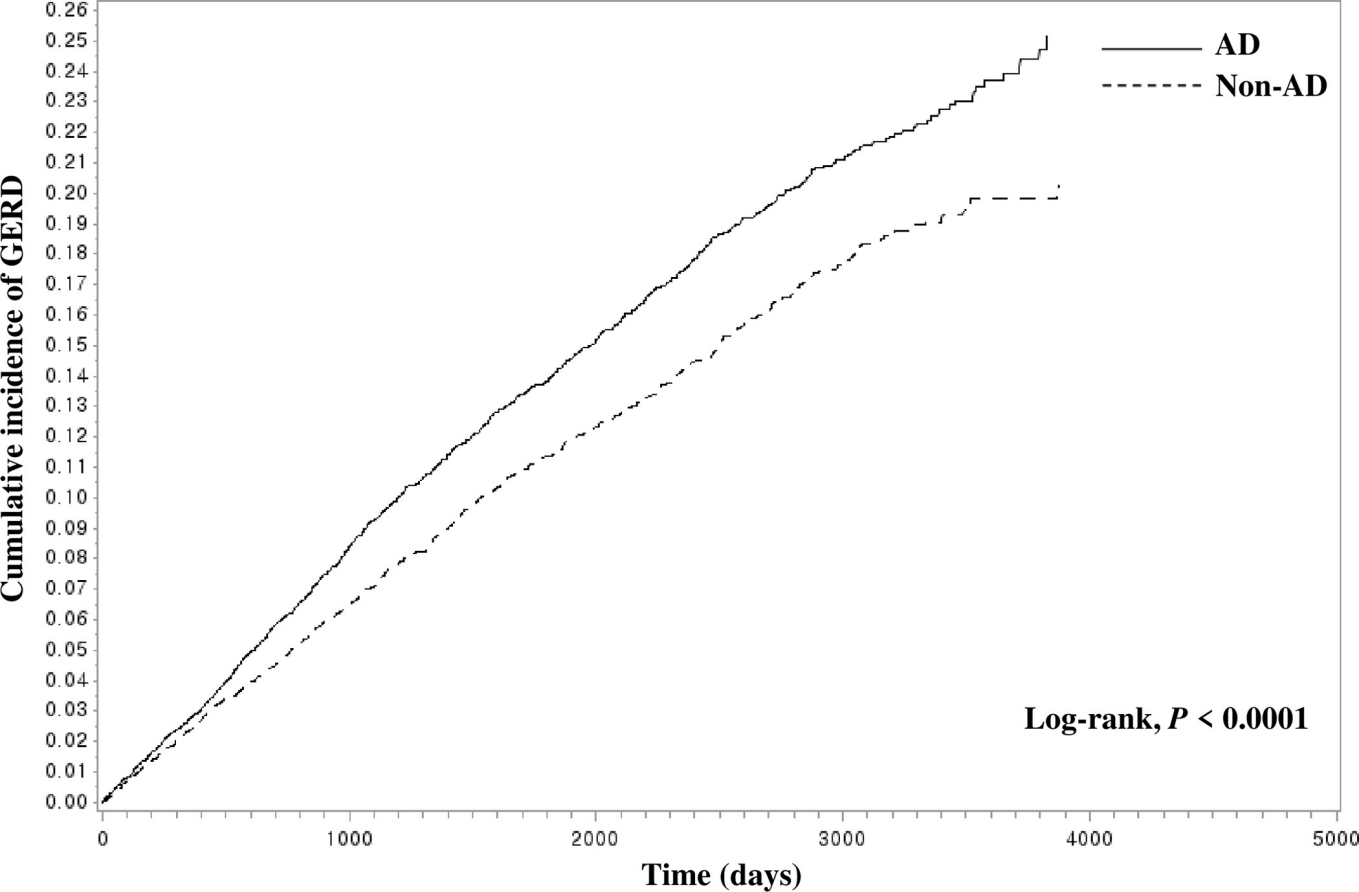
Cumulative incidence of GERD in patients with (solid line) and without AD (dashed line) over time. AD, atopic dermatitis; GERD, gastroesophageal reflux disease.

Patients with AD had a 15% greater risk of developing GERD after full adjustment for potential covariates than the matched controls (adjusted HR, 1.15; 95% CI, 1.06–1.26; Table 2). The cumulative incidence of study outcomes over time in adults with and without AD demonstrated a higher incidence of GERD in adults with AD than in those without AD (log-rank, *P* < 0.0001; Fig 2).

**Table 2:** Risk of GERD in patients diagnosed with AD.

	No.	Event (%)	Crude HR (95% CI)	*P* value	Adjusted HR (95% CI)^a^	*P* value
Total						
AD	9,164	1,130 (12.33)	1.23 (1.13–1.34)	<0.0001	1.15 (1.06–1.26)	0.0013
Non-AD	9,164	949 (10.36)	Reference		Reference	

AD, atopic dermatitis; CI, confidence interval; GERD, gastroesophageal reflux disease; HR, hazard ratio.

^a^Adjusted for age, sex, household income, region of residence, disability, Charlson comorbidity index, smoking status, body mass index, co-medications, and baseline year.

The difference in the risk of developing GERD according to the severity of AD was not statistically significant among patients with AD (S1 Table).

### Subgroup analyses

The sex-stratified analysis revealed a 15% and 17% higher risk of GERD in male (adjusted HR, 1.15; 95% CI, 1.01–1.30) and female (adjusted HR, 1.17; 95% CI, 1.03–1.26) with AD than that in those without AD, respectively (Table 3).

**Table 3:** Subgroup analysis of GERD risk based on sex.

	No.	Event (%)	Crude HR (95% CI)	*P* value	Adjusted HR (95% CI)^a^	*P* value
Male						
AD	4,816	532 (11.05)	1.24 (1.09–1.40)	0.0009	1.15 (1.01–1.30)	0.0343
Non-AD	4,815	444 (9.22)	Reference		Reference	
Female						
AD	4,348	598 (13.75)	1.22 (1.08–1.37)	0.0012	1.17 (1.03–1.26)	0.0120
Non-AD	4,349	505 (11.61)	Reference		Reference	

AD, atopic dermatitis; CI, confidence intervals; GERD, gastroesophageal reflux disease; HR, hazard ratio.

^a^Adjusted for age, sex, household income, region of residence, disability, Charlson comorbidity index, smoking status, body mass index, co-medications, and baseline year.

The subgroup analysis according to the age group revealed a constant significant increased risk of GERD after adjusting for potential covariates, except for the 60 years and older group: age group 20–39 years (adjusted HR, 1.40; 95% CI, 1.12–1.76), age group 40–59 years (adjusted HR, 1.16; 95% CI, 1.03–1.31), and ≥60 years of age group (adjusted HR, 1.03; 95% CI, 0.88–1.20) (Table 4).

**Table 4:** Subgroup analysis of GERD risk based on age.

	No.	Event (%)	Crude HR (95% CI)	*P* value	Adjusted HR (95% CI)^a^	*P* value
20–39 years						
AD	2,557	191 (7.47)	1.51 (1.21–1.89)	0.0003	1.40 (1.12–1.76)	0.0030
Non-AD	2,557	129 (5.04)	Reference		Reference	
40–59 years						
AD	4,504	610 (13.54)	1.25 (1.11–1.41)	0.0002	1.16 (1.03–1.31)	0.0125
Non-AD	4,504	501 (11.12)	Reference		Reference	
60 years and older						
AD	2,103	329 (15.64)	1.08 (0.92–1.26)	0.3474	1.03 (0.88–1.20)	0.7537
Non-AD	2,103	319 (15.17)	Reference		Reference	

AD, atopic dermatitis; CI, confidence intervals; GERD, gastroesophageal reflux disease; HR, hazard ratio.

^a^Adjusted for age, sex, household income, region of residence, disability, Charlson comorbidity index, smoking status, body mass index, co-medications, and baseline year.

### Sensitivity analyses

All sensitivity analysis results using different AD or GERD definitions were consistent with the results of the main analysis (S2–S4 Tables).

## Discussion

This nationwide study using well-established cohort data from South Korea evaluated the subsequent risk of GERD in newly diagnosed adult patients with AD. AD and GERD have a high prevalence and can coexist independently, making the assessment of a direct relationship between them difficult. Therefore, this study longitudinally evaluated the risk of developing GERD after AD diagnosis and identified a significant association between AD and GERD development in adults in both crude and adjusted models.

The subsequent increased risk of GERD in patients diagnosed with AD remained significant in subgroup analyses by sex or age groups under 60 years old as well as in all sensitivity analyses. Exceptionally, the risk was not significant in the subgroup above 60 years of age. Given that GERD is the most common upper gastrointestinal disease that frequently occurs in the elderly [26], one possible explanation for this finding is that the intrinsically high incidence of GERD in the elderly may mitigate the impact of AD and its statistical significance.

Although the relationship between AD and GERD as well as their underlying mechanisms need to be further investigated, several possible factors link AD with the development of GERD. One possible explanation is the inappropriate modulation of the lower esophageal sphincter (LES). Gastroesophageal reflux occurs pathologically in GERD and is related to a transient loss of LES pressure, which is controlled by the activity of neurotransmitters released by the vagus nerve and enteric nervous system stimulation [27, 28]. Interestingly, psychiatric symptoms, such as depression or anxiety, which are commonly observed in patients with AD, are associated with an autonomic dysbalance with relatively decreased vagal activity [29]. In addition, histamine, which plays an important role in the pathophysiology of AD and whose plasma level increases during acute exacerbations, may promote the pathogenesis of GERD through the modulation of LES contractions [30, 31]. Another possible factor is protease-activated receptor-2 (PAR2) activation. PAR2 activation is associated with inflammation, pruritus, and skin barrier regulation, which are hallmarks of AD [32]. Increased PAR2 expression is linked to high interleukin-8 levels within the mucosa, which allows inflammatory cells to be recruited into the mucosa [33]. These observations support the concept that GERD may be caused by an immune-mediated process in AD. Lastly, the same risk factor, such as sleep disturbance, can be considered. Sleep disturbance is one of the most burdensome symptoms reported in adults with AD. Several studies have reported that the prevalence of sleep disturbances in adult patients with AD is as high as 33%–87.1% [34–36]. Meanwhile, sleep disturbance also appears to be associated with GERD. Although the exact mechanism has not yet been established, it is speculated that the relationship between sleep disturbance and GERD is bidirectional [37, 38].

This study had several strengths. First, to the best of our knowledge, this study was the first to primarily focus on the direct temporal relationship between AD and GERD in a general population of adults. Second, this study utilized the NHIS-NSC database of South Korea, which is representative of the entire population and provides data on healthcare utilization from all settings in South Korea. However, it should be noted that due to inherent limitations in the claims database, patients with mild symptoms who treated themselves at home without seeking medical care might be excluded from the research. Third, this study identified AD or GERD by considering both the diagnostic code and treatment medication. In this study, patients with AD were defined as those who had at least one diagnostic code and were prescribed AD-related medications on the same day. Studies that have assessed the specificity or sensitivity of methods for defining AD using diagnostic codes supported this patient classification method. When employing one AD code and two AD-related treatment codes, a study in the UK found that the positive predictive value was 86% [39]. In a different study conducted in Korea, it was discovered that there was no difference in the specificity of the groups defined by the two diagnostic codes alone or by combining the diagnostic code and AD laboratory code [40]. Additionally, although many studies have defined AD based on self-report questionnaires, these questionnaire-based methods for identifying adults with AD have not been validated and are known to have the potential to introduce selection bias [41]. Meanwhile, GERD was defined as a case in which a patient was diagnosed with GERD twice or more and was prescribed GERD-related medications for more than 2 weeks. This is because there is insufficient evidence to support standard methods of defining GERD in studies using claims databases. And in Korea, GERD-related medications such as histamine 2-receptor antagonists, proton pump inhibitors, and potassium competitive gastric acid blockers are frequently prescribed not only for GERD but also for other diseases. To overcome this and reduce the possibility of misclassification, the number of diagnostic codes was strengthened when defining GERD. The validity of this study design is supported by the results of sensitivity analyses employing different definitions of AD or GERD, consistent with the main findings. Nevertheless, misclassification should be considered when interpreting study results due to inherent limitations such as coding inaccuracy (over-coding, under-coding, or miscoding) and a lack of disease specificity of claims databases. Fourth, this study had a sufficient follow-up time to investigate the association between AD and GERD and performed PSM and several sensitivity analyses to reduce the effects of residual confounding and bias.

Despite these strengths, some limitations should be considered when interpreting the study results. First, the potential for misclassification remains although patients newly diagnosed with AD were defined using more stringent criteria than other previous studies based solely on self-report questionnaires or diagnostic codes. For example, childhood-onset AD that persists or recurs into adulthood may be enrolled in the study. However, a recent systematic review of 46 studies evaluating the incidence and risk factors of AD revealed a less common AD persistence. They reported that only 5% of cases had no observed clearance after 20 years of follow-up [2]. Even if there was a problem of misclassification because childhood AD was included in the control and patient groups, it would have acted in the direction of weakening the association between AD and GERD as a nondifferential misclassification. This study excluded cases diagnosed with AD between 2002 and 2004 from the study group to exclude recurrent or persistent cases (3-year washout period). Therefore, including persistent or recurrent AD cases is unlikely. In addition, various sensitivity analyses were performed to overcome this limitation, and all the results were consistent with the main results. Second, in this study, other allergic diseases that could confound the effects of AD were not considered when selecting subjects. Hence, patients with other allergic diseases were included in the case and control groups, which might have contributed to the increased risk of AD. However, the effects of nondifferential misclassification would have resulted in a bias toward the null [42]. Third, possible residual confounding factors including dietary, personal lifestyle, duration of topical corticosteroid treatment or applied skin site, or other environmental factors could not be considered in the statistical analysis because of the inherent claims database limitations. However, this study attempted to adjust for all available factors, including household income and region of residence, as well as demographic factors, such as age, sex, smoking status, CCI score, BMI, and co-medications. Fourth, unlike previous research that found that the severity of atopic disease increased the risk of comorbidity, there was no significant difference in the risk of GERD between the severity groups in our study. As patients who had ever received oral corticosteroids or immunosuppressants were considered as having severe AD in this study, this may be attributable to the unintended inclusion of nonsevere AD in severe AD. More prospective studies are required to clarify this since standards for defining severe AD have not been established in studies employing claims databases. Lastly, causation could not be demonstrated, although the study results suggest the role of AD in GERD development.

In conclusion, this nationwide cohort study revealed that AD development was associated with an increased risk of subsequent GERD in Korean adults. The results of this study contribute to our understanding of the risk of GERD in adult patients with AD and suggest that appropriate efforts, such as proper drug selection or lifestyle management considering the risk of GERD, may be required when treating AD.

## Supporting information

S1 Table Risk of GERD in patients diagnosed with AD according to the severity. (PDF)

S2 Table Sensitivity analysis according to the different definition of AD. (PDF)

S3 Table Sensitivity analysis according to the different definition of GERD. (PDF)

S4 Table Sensitivity analysis according to including antacids in the drugs defining GERD. (PDF)
