# Supplementary material for: Atopic dermatitis and risk of gastroesophageal reflux disease: A nationwide population-based study
Source: PLoS One. 2023 Feb 17;18(2):e0281883. doi: 10.1371/journal.pone.0281883 (PMC9937456; doi:10.1371/journal.pone.0281883)
Supplement: S1 Table — (PDF) [file pone.0281883.s001.pdf]

S1 Table. Risk of GERD in patients diagnosed with AD according to the severity.

	No.	Event (%)	Crude HR (95% CI)	*P* value	Adjusted HR (95% CI)[a]	*P* value
Total						
Severe	4,297	528 (12.29)	1.04 (0.92-1.18)	0.4961	1.03 (0.91-1.16)	0.6506
Non-severe	4,297	511 (11.89)	Reference		Reference	

Cox proportional hazard models were used to estimate the risk of GERD among severe AD patients compared to nonsevere AD patients.

AD, atopic dermatitis; CI, confidence intervals; GERD, Gastroesophageal reflux disease; HR, hazard ratio.

[a]Adjusted for age, sex, household income, region of residence, disability, Chralson comorbidity index, smoking status, body mass index, co-medications, and baseline year.
