# Supplementary material for: Atopic dermatitis and risk of gastroesophageal reflux disease: A nationwide population-based study
Source: PLoS One. 2023 Feb 17;18(2):e0281883. doi: 10.1371/journal.pone.0281883 (PMC9937456; doi:10.1371/journal.pone.0281883)
Supplement: S2 Table — (PDF) [file pone.0281883.s002.pdf]

S2 Table. Sensitivity analysis according to the different definition of AD[a].

	No.	Event (%)	Crude HR (95% CI)	*P* value	Adjusted HR (95% CI)[b]	*P* value
Total						
AD	6,745	836 (12.39)	1.23 (1.11-1.36)	<.0001	1.15 (1.04-1.28)	0.0054
Non-AD	6,745	702 (10.41)	Reference		Reference	

Cox proportional hazard models were used to estimate the risk of GERD among participants with AD compared to those without AD.

AD, atopic dermatitis; CI, confidence intervals; GERD, Gastroesophageal reflux disease; HR, hazard ratio.

[a]A definition of AD is two or more prescriptions of AD drugs with a primary or secondary diagnosis of AD (International Classification of Disease 10th code L20).

[b]Adjusted for age, sex, household income, region of residence, disability, Charlson comorbidity index, smoking status, body mass index, co-medications, and baseline year.
