# Supplementary material for: Atopic dermatitis and risk of gastroesophageal reflux disease: A nationwide population-based study
Source: PLoS One. 2023 Feb 17;18(2):e0281883. doi: 10.1371/journal.pone.0281883 (PMC9937456; doi:10.1371/journal.pone.0281883)
Supplement: S3 Table — (PDF) [file pone.0281883.s003.pdf]

S3 Table. Sensitivity analysis according to the different definition of GERD[a].

	No.	Event (%)	Crude HR (95% CI)	*P* value	Adjusted HR (95% CI)[b]	*P* value
Total						
AD	9,854	819 (8.31)	1.23 (1.11-1.36)	<0.0001	1.15 (1.04-1.28)	0.0059
Non-AD	9,854	682 (6.92)	Reference		Reference	

Cox proportional hazard models were used to estimate the risk of GERD among participants with AD compared to those without AD.

AD, atopic dermatitis; CI, confidence intervals; GERD, Gastroesophageal reflux disease; HR, hazard ratio.

[a]A definition of GERD is three or more diagnosis codes (International Classification of Disease 10th code K21) and prescriptions of GERD drugs for more than 3 weeks.

[b]Adjusted for age, sex, household income, region of residence, disability, Charlson comorbidity index, smoking status, body mass index, co-medications, and baseline year.
