# Supplementary material for: Atopic dermatitis and risk of gastroesophageal reflux disease: A nationwide population-based study
Source: PLoS One. 2023 Feb 17;18(2):e0281883. doi: 10.1371/journal.pone.0281883 (PMC9937456; doi:10.1371/journal.pone.0281883)
Supplement: S4 Table — (PDF) [file pone.0281883.s004.pdf]

S4 Table. Sensitivity analysis according to including antacids in the drugs defining GERD[a].

	No.	Event (%)	Crude HR (95% CI)	*P* value	Adjusted HR (95% CI)[b]	*P* value
Total						
AD	6,230	2,062 (33.10)	1.38 (1.29-1.47)	<0.0001	1.33 (1.25-1.42)	<0.0001
Non-AD	6,230	1,629 (26.15)	Reference		Reference	

Cox proportional hazard models were used to estimate the risk of GERD among participants with AD compared to those without AD.

AD, atopic dermatitis; CI, confidence intervals; GERD, Gastroesophageal reflux disease; HR, hazard ratio.

[a]Antacids were additionally included in the drugs defining GERD.

[b]Adjusted for age, sex, household income, region of residence, disability, Charlson comorbidity index, smoking status, body mass index, co-medications, and baseline year.
